# A Case Series and Literature Review of Subacute Infective Endocarditis: A Clinical Challenge

**DOI:** 10.7759/cureus.35997

**Published:** 2023-03-10

**Authors:** Santiago Callegari, Alfredo Cabrera, Laura Mejia, Carlos A Sanchez

**Affiliations:** 1 Cardiology, Beth Israel Deaconess Medical Center, Harvard Medical School, Boston, USA; 2 Cardiology, Hospital Universitario Fundación Santa Fe de Bogotá, Bogota, COL; 3 Cardiology, Universidad el Bosque, Bogota, COL; 4 Medicine, Universidad de los Andes, Bogota, COL

**Keywords:** gemella sanguinis, parvimonas micra, mitral valve, aortic valve, granulicatella adiacens, endocarditis, case report

## Abstract

Endocarditis is a life-threatening, relatively rare disease caused by an infection of the endocardial epithelium of the heart. Its clinical presentation is highly variable, depending on whether it presents acutely, subacutely, or chronically. Also, the wide array of causal etiologies and pathogens makes its diagnosis and treatment complex and challenging. The main etiological agents are S*taphylococci *and S*treptococci*, while fastidious microorganisms are infrequent agents of this pathology. Advancements in the identification of microorganisms with novel molecular techniques have revealed new previously unidentified pathogens. Despite their low frequency, these fastidious pathogens are highly relevant, as they have been associated with a higher rate of complications and mortality. Therefore, it is necessary to be aware of the wide array of clinical presentations and important considerations for the management of patients with subacute endocarditis with atypical microorganisms. In this article, we present a case series involving three different clinical presentations of subacute endocarditis with fastidious microorganisms, which required extensive medical management and surgical valve repair with favorable and unfavorable outcomes. We also engage in a review of the literature on their microbiology, diagnosis, and treatment.

## Introduction

Infective endocarditis is a relatively infrequent disease, with a reported crude incidence ranging from 1.5 to 11.6 cases per 100,000 person-years; it is associated with high morbidity and mortality worldwide [1]. In South America, endocarditis is mainly caused by *Streptococcus* species, *Staphylococcus aureus*, and blood culture-negative microorganisms [2]. The steady advancements in the laboratory and molecular identification techniques have revealed previously unidentified pathogens, which have also been associated with a higher complication rate and significant mortality [3]. Our case series highlights the wide scope of different clinical presentations and describes pathogens rarely reported in the literature. We report three cases of subacute endocarditis caused by uncommon microorganisms, characterized by a challenging clinical presentation and management. The article also provides a review of the literature concerning microbiology and special considerations related to these cases. We followed the appropriate case report guidelines (CARE) for adequately describing the cases and informed consent was obtained from all three patients prior to accessing medical records as well as the publication of the report [4].

## Case presentation

Case 1

The patient was a 78-year-old woman with a history of immunosuppression due to long-standing treatment for rheumatoid arthritis with infliximab. She presented with complaints of malaise, fatigue, and low-grade fever for 10 days, which had exacerbated over the three days prior to admission, accompanied by atypical chest pain, dyspnea, and deterioration of functional class to the New York Heart Association (NYHA) classification class II. Her past medical history included hypertension, dyslipidemia, and rheumatoid arthritis in remission. Acute coronary syndrome was initially ruled out based on electrocardiogram findings and cardiac biomarkers, and blood cultures were taken given the febrile syndrome and symptoms, due to the suspicion of endocarditis. A transthoracic echocardiogram was performed, which ruled out contractility disorders and showed an image suggestive of a vegetation of 1.1 cm in the posterior leaflet of the mitral valve. The evaluation was completed with a transesophageal echocardiogram with consistent findings. The main diagnostic suspicion was possible infective endocarditis of the native valve, and treatment was started with vancomycin and ceftriaxone.

On the second day of hospitalization, the patient experienced altered sensory perception, disorientation, and visual hallucinations. A brain MRI was ordered due to a possible septic embolism. The images revealed multiple areas of acute and subacute infarcts involving the anterior and posterior as well as supra and infratentorial vascular territories. In the following days, the patient's neurological abnormalities slightly improved. On the sixth day of hospitalization, blood cultures became positive, isolating *Parvimonas micra*, thereby fulfilling the criteria for definitive endocarditis. Based on the antibiogram, antibiotic treatment was adjusted, changing to crystalline penicillin 4,000,000 IU every four hours. On the 11th day of hospitalization, the patient had a second episode of neurological deterioration, involving disorientation, which raised suspicion of cerebellar ataxia. The brain MRI was repeated, which confirmed new areas of cerebellar infarction.

**Figure 1:**
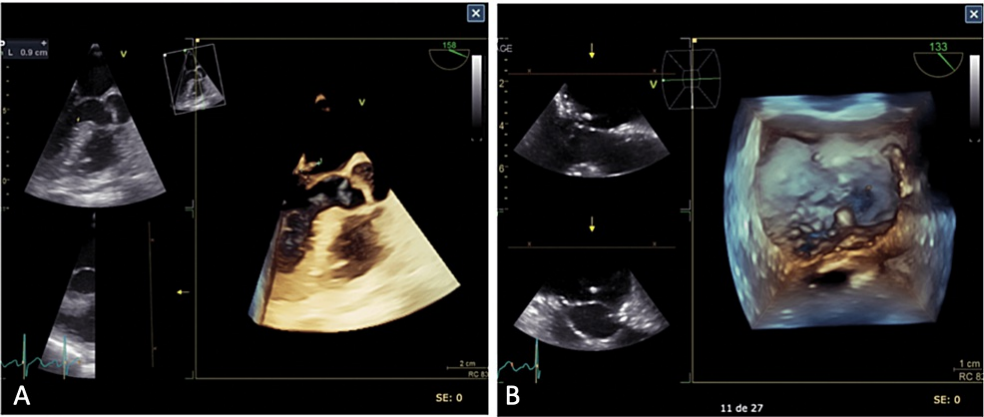
Transesophageal echocardiogram revealing Parvimonas micra vegetation 1A: 3D transesophageal ultrasound showing vegetation on the posterior mitral leaflet with marked mobility towards the auricular surface. 1B: Same sonography with a different projection revealing vegetation on P2

Due to the recurrence of the embolic event, a new transesophageal echocardiogram was performed (Figure 1), which showed an abscess on the posterior leaflet at P2, with two filamentous images of 0.9 mm, one on the atrial side and the other on the ventricular side, as well as moderate mitral insufficiency. Due to complications related to infective endocarditis, the patient was a candidate for mitral valve replacement per guideline recommendations. However, severe calcification of the mitral valve plane with the involvement of the subvalvular apparatus was documented, limiting her suitability for the procedure. Hence, it was decided to continue with medical treatment only, which was completed without other abnormalities. Video 1 depicts the 3D transesophageal ultrasound procedure.

**Video 1:** 3D transesophageal ultrasound revealed vegetation due to Parvimonas micra subacute endocarditis The vegetation was located in the posterior mitral leaflet, with marked mobility toward the auricular surface

Case 2

A 50-year-old male patient, previously healthy and athletic, presented to the emergency department 45 days after undergoing a rotator cuff surgery and with iron deficiency anemia under study. He was admitted to the hematology department of Fundación Santa Fe de Bogotá for a one-month evolution of subjective fever, nocturnal diaphoresis, weight loss of 10 kilograms in one month, asthenia, and adynamia. Additionally, he reported insomnia and nocturnal hallucinations. He had previously consulted through telemedicine, and at that time, opioid withdrawal had been considered due to the suspension of tapentadol used for analgesia in the postoperative period. He had been started on alprazolam, which did not lead to any improvement. Hence, he decided to present to the hospital. As for his background, he had experienced a rupture of the supraspinatus tendon and had iron deficiency anemia under study for several years. His family history was positive for prostate cancer and metastatic melanoma on his mother's side.

During the hematology consult, on physical examination, the patient was found to be in a deteriorated general condition by his primary care physician. On auscultation, a diastolic decrescendo murmur in the aortic focus and a holosystolic murmur in the mitral focus of recent origin were found. There were no other relevant findings on physical examination. Therefore, the patient was referred to the emergency department, where the same findings were reported. The patient was immediately started on ampicillin and gentamicin.

**Figure 2:**
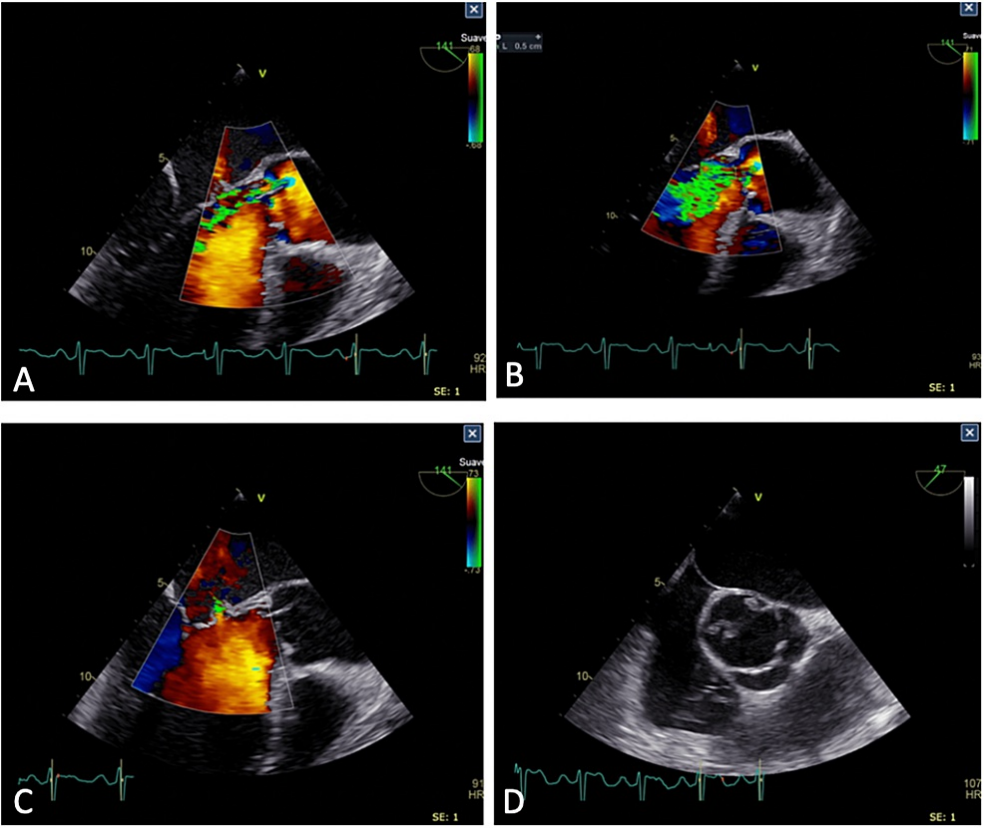
Transthoracic echocardiogram revealing abnormalities consistent with infective endocarditis. 2A, 2B: Evidence of marked aortic insufficiency with two jets (one eccentrical and one centrical). 2C: Evident perforation as a consequence of the abscess, which resulted in an eccentric jet. 2D: 2D echocardiogram showing vegetation on the bicuspid aortic valve

In the initial blood tests, there was no presence of leukocytosis (8600 cells/milliliter) or neutrophilia (6600 cells/milliliter). In the erythropoietic cell line, there was the presence of mild normocytic anemia (hemoglobin: 11.1 mg/dl) with altered ferrokinetics [total iron: 19 ug/dL, ferritin: 531 ug/L, total iron binding capacity (TIBC): 245.1 ug/dL]. The transthoracic ultrasound evidenced a dilated left ventricle, an ejection fraction of 47%, and aortic vegetation of 1.1 x 0.6 cm in its largest diameter associated with aortic insufficiency (Figure 2, Video 2). Additionally, there was the presence of mitral insufficiency with thickened anterior leaflet and an anechoic image in its interior, with an image of perforation that revealed the presence of a jet with severe insufficiency (Video 3). The findings were confirmed by transesophageal ultrasound, which showed a bivalve aortic valve with rupture of the non-coronary leaflet, abscess, and rupture of the anterior mitral leaflet. Initially, blood cultures reported *Granulicatella adiacens* sensitive to vancomycin, and hence new management with this antibiotic was started. When surgery was indicated, the patient was referred to cardiovascular surgery for bivalvular replacement, which was successfully performed and the patient's condition subsequently improved.

**Video 2:** Transthoracic echocardiogram showing vegetations on the bicuspid aortic valve (left) and mitral valve (right) caused by subacute endocarditis due to Granulicatella adiacens

**Video 3:** Transthoracic echocardiogram in Doppler mode showing marked mitral insufficiency caused by subacute endocarditis due to Granulicatella adiacens

Case 3

A 79-year-old male patient with a previous history of mild kidney dysfunction presented with dysarthria, vertigo, and motor aphasia associated with unquantified fever. An emergency brain MRI revealed multiple acute infarcts. Ischemic stroke of cardioembolic appearance was considered. Based on FLAIR and DWI MRI findings, it was understood that the stroke had occurred more than six hours ago, and hence reperfusion was not performed. As part of the etiological evaluation, a transthoracic echocardiogram was performed, which was normal. However, due to strong clinical suspicion of infective endocarditis, the patient underwent a transesophageal echocardiogram, which revealed mobile lesions on the aortic valve: one on the right coronary leaflet measuring 1.03 x 0.5 cm and another on the left coronary leaflet measuring 0.54 x 0.76 cm, with mild insufficiency. Based on this finding, blood cultures were taken and treatment was started with ceftriaxone and linezolid due to the suspicion of infective endocarditis. In order to better characterize the lesions and search for potential complications of endocarditis as well as to perform coronary stratification, a coronary angiotomography was performed without complications. It revealed vegetations measuring 9.7 x 9.5 x 8 mm, without any other significant findings. The blood culture report showed the growth of *Gemella sanguinis*. The antibiotic treatment was changed to gentamicin and ceftriaxone. On day seven of the treatment, the transesophageal echocardiogram was repeated and showed the persistence of vegetations, with changes of valvular insufficiency in progression. Hence, the patient was considered a candidate for surgical treatment, which he refused. Prior to discharge, significant periodontal disease was found, which was treated during hospitalization. The patient subsequently completed antibiotic treatment at home without any further complications.

The main laboratory findings of case 1 and case 2 are shown in Table 1 in the Appendix.

## Discussion

Gram-positive anaerobic cocci are not the microorganisms we most expect to find in cases of infective endocarditis. *Parvimonas micra* is a gram-positive coccus, commensal to the normal oral flora in humans [5], which has been implicated mainly in infections of the oral cavity (mainly mucositis, sialoadenitis, and parotitis), soft tissue, and bone [6]. Similarly, *Granulicatella adiacens* is another gram-positive coccus, which is classified within the *streptococci* of variable nutrition [7]. Like *Parvimonas micra*, this bacterium is mainly found in the oral cavity and has been associated with a variety of infections such as pancreatic abscesses, conjunctivitis, sepsis, and endocarditis. However, some case reports have described the pathogenic potential of both bacteria in endocarditis [7]. Finally, *Gemella sanguinis* is a gram-positive, catalase-negative, facultatively anaerobic coccus, which is also part of the normal microbiota of the oropharynx [8].

According to data from the last decade, endocarditis caused by anaerobic gram-positive cocci accounts for 1.3% of all cases [9], with *Propionibacterium acnes* and *Bacteroides fragilis* being the most frequently isolated agents, while *Parvimonas micra* accounts for approximately 6% of cases. *Granulicatella adiacens* has been found in a similar proportion (approximately 5-8%), and *Gemella sanguinis* accounts for 4.6% of cases [9].

The gateway for bacteremia and subsequent endocarditis due to anaerobic microorganisms is usually the respiratory, gastrointestinal, or urinary tract. They have also been associated with dental procedures, mainly periodontal surgery (88%), dental extractions (60%), and tonsillectomy (35%) [10]. However, a history of interventional periodontal disease was found in only one of the three cases described. This phenomenon can be explained by the fact that bacteremia is reported in up to 26% of cases after simple activities such as brushing your teeth [9].

The clinical presentation of endocarditis due to these microorganisms is usually subtle and sub-acute, which often leads to delays in diagnosis. The main manifestations include constitutional symptoms of several months of evolution including anorexia, weight loss, and fever, followed by heart failure or embolisms. Classic signs such as Roth's spots or Osler's nodules are infrequent [11]. The rate of complications and mortality is higher with these atypical microorganisms [8,12] compared to other types of endocarditis. This higher frequency of complications is believed to be related to the subacute presentation or even special microbiological conditions of these microorganisms. For example, the production of heparinases by *Bacteroides fragilis* [11], or the greater affinity of *Granulicatella adiacens* for the valvular endothelium [8], the latter resulting in a similar mechanism as reported for *Streptococcus mutans* for increased collagen and laminin-binding [13,14]. The infection commonly involves the aortic and mitral valves, as reported in up to 64% of cases. Mortality reported in other case series of *Granulicatella adiacens *infection averages 17-27%, and the development of heart failure occurs in 30% of patients [15].

Two tests are fundamental for reaching the diagnosis, both of which have important considerations: 

1. Blood culture: in this setting, it has three limitations: low sensitivity if standard culture methods are used, the slow growth rate of microorganisms, and high rate of polymicrobial cultures (up to 24% of cultures show polymicrobial findings in these cases) [9,8,13]. In the specific case of *Granulicatella adiacens*, another limitation is the considerable difficulty in obtaining successful growth in culture. It requires, above all, supplementation with pyridoxine for its growth [7]. Endotracheal tube culture can be considered in the setting of chronically bedridden ICU patients apart from blood culture.

2. Echocardiogram: this is a fundamental test for reaching a diagnosis. It should be performed early since its delay can lead to valve destruction, embolization, and subsequent valve surgery [16].

Regarding the treatment of infective endocarditis, the European Society of Cardiology recommends the use of penicillin G 12-18 million units per day, amoxicillin 100-200 mg/kg/day, or ceftriaxone 2 g/day IV for four weeks [17]. However, frequent fatalities due to the disease have been reported after using the treatment recommended by American and European guidelines. Despite in vitro effectiveness, treatment failure occurs in up to 41% of cases, with a relapse rate of 17% and almost 27% of patients require a prosthetic valve [18]. Nutritional variant *streptococci* have developed a tendency to penicillin resistance in recent years, with only 40% of *Granulicatella* isolates being reported as penicillin-sensitive, and more than 50% reporting intermediate ranges of sensitivity [17]. Standard indications for surgery should also be taken into account, which include severe heart failure, severe valve dysfunction, prosthetic valve infection, invasion beyond the valve leaflets, recurrent systemic embolization, large mobile vegetations, or persistent sepsis despite adequate antibiotic therapy for more than five to seven days [19].

## Conclusions

Subacute endocarditis represents a challenge for the clinician in terms of both diagnosis and treatment. However, it is necessary to take it into consideration promptly since a delay in diagnosis results in a considerable increase in complications and mortality rates. The improvement in the typing of microorganisms in recent years requires the clinical cardiologist to be up to date with the characteristics and considerations of these new microorganisms in order to manage them appropriately, which requires a prompt diagnosis, early administration of antibiotics, and appropriate decision-making related to complications and surgery when indicated.

## Appendices

**Table 1:** Main laboratory results of case 1 and case 2

		Case 1 *(Granulicatella adiacens)*	Case 2 *(Parvimonas micra)*
Blood biochemistry results			
	Hemoglobin (mg/dl)	11.1	13.0
	Hematocrit (%)	34.7	40.3
	Leucocytes (cells/uL)	8600	9100
	Neutrophils (cells/uL)	6600	7900
	Platelets (cells/uL)	213,000	215,000
	Lactate dehydrogenase (LDH)	177	
Liver function tests			
	Aspartate aminotransferase (ALT)	40	48
	Alanine aminotransferase (AST)	61	37
	Alkaline phosphatase (ALP)	205	102
	Total bilirrubin (mg/dl)	0.72	0.52
Kidney function			
	Creatinine (mg/dl)	0.95	0.51
	Sodium	137	131
	Potassium	4.5	3.6
Ferrokinetics			
	Ferritin	531	73.8
	Total iron	19	
	Total Iron binding capacity (TIBC)	245.1	
Others			
	Vitamin B12	512	
	Thyroid-stimulating hormone (TSH)	3.1	
	Erythrocyte sedimentation rate (ESR)	26	65
	C-reactive protein (CRP)	6.4	
